# Warfarin anticoagulation management during the COVID-19 pandemic: The role of internet clinic and machine learning

**DOI:** 10.3389/fphar.2022.933156

**Published:** 2022-09-26

**Authors:** Meng-Fei Dai, Shu-Yue Li, Ji-Fan Zhang, Bao-Yan Wang, Lin Zhou, Feng Yu, Hang Xu, Wei-Hong Ge

**Affiliations:** ^1^ Department of Pharmacy, Nanjing Drum Tower Hospital, The Affiliated Hospital of Nanjing University Medical School, Nanjing, China; ^2^ School of Basic Medicine and Clinical Pharmacy, China Pharmaceutical University, Nanjing, China; ^3^ Department of Pharmacy, Shanxi Province Cancer Hospital, Taiyuan, China; ^4^ Nanjing Foreign Language School, Nanjing, China

**Keywords:** telemedicine, COVID-19, warfarin, internet, machine learning, anticoagulation quality

## Abstract

**Background:** Patients who received warfarin require constant monitoring by hospital staff. However, social distancing and stay-at-home orders, which were universally adopted strategies to avoid the spread of COVID-19, led to unprecedented challenges. This study aimed to optimize warfarin treatment during the COVID-19 pandemic by determining the role of the Internet clinic and developing a machine learning (ML) model to predict anticoagulation quality.

**Methods:** This retrospective study enrolled patients who received warfarin treatment in the hospital anticoagulation clinic (HAC) and “Internet + Anticoagulation clinic” (IAC) of the Nanjing Drum Tower Hospital between January 2020 and September 2021. The primary outcome was the anticoagulation quality of patients, which was evaluated by both the time in therapeutic range (TTR) and international normalized ratio (INR) variability. Anticoagulation quality and incidence of adverse events were compared between HAC and IAC. Furthermore, five ML algorithms were used to develop the anticoagulation quality prediction model, and the SHAP method was introduced to rank the feature importance.

**Results:** Totally, 241 patients were included, comprising 145 patients in the HAC group and 96 patients in the IAC group. In the HAC group and IAC group, 73.1 and 69.8% (*p* = 0.576) of patients achieved good anticoagulation quality, with the average TTR being 79.9 ± 20.0% and 80.6 ± 21.1%, respectively. There was no significant difference in the incidence of adverse events between the two groups. Evaluating the five ML models using the test set, the accuracy of the XGBoost model was 0.767, and the area under the receiver operating characteristic curve was 0.808, which showed the best performance. The results of the SHAP method revealed that age, education, hypertension, aspirin, and amiodarone were the top five important features associated with poor anticoagulation quality.

**Conclusion:** The IAC contributed to a novel management method for patients who received warfarin during the COVID-19 pandemic, as effective as HAC and with a low risk of virus transmission. The XGBoost model could accurately select patients at a high risk of poor anticoagulation quality, who could benefit from active intervention.

## Introduction

Coronavirus disease 2019 (COVID-19) is a respiratory infection spreading around the world sharply, with a high rate of mortality (Wu and McGoogan, 2020). It has been recognized as a pandemic by the World Health Organization (World Health Organization, 2021). On 23 January 2020, China initiated the Level I response to public health incidents nationwide, which is the highest one (Jiang et al., 2022), and slowed the spread of COVID-19 effectively. However, social distancing and stay-at-home orders, which were adopted to avoid the spread of COVID-19, posed unique challenges for patients on medications, requiring continued monitoring by clinic staff, such as warfarin treatment (Kish and Lekic, 2021).

The vitamin K antagonist warfarin is an anticoagulant drug widely used in thromboprophylaxis and treatment. Warfarin has a narrow therapeutic window and wide variability in dose–response (Gu et al., 2018). It is necessary to frequently monitor the International Normalized Ratio (INR) and adjust the dosage accordingly to maintain the INR within the therapeutic range, which can ensure the effectiveness and safety of chronic warfarin treatment (Gu et al., 2019). Clinical pharmacists provide professional anticoagulation management services (AMSs) in hospital anticoagulation clinics (HACs) (Holbrook et al., 2012), including INR testing, dose adjustment, and medication education, which are associated with better anticoagulation quality than usual physician care (Ahmed et al., 2017; Manzoor et al., 2017). Unfortunately, the COVID-19 pandemic has made it difficult for patients to visit anticoagulation clinics to obtain AMSs (Gong et al., 2020; Zhang, 2020). Previous studies have shown a significant increase in adverse events of warfarin treatment during COVID-19 stringency measures (Vriz et al., 2021). Therefore, measures should be taken to improve the anticoagulation quality of patients during the COVID-19 pandemic.

For chronic disease management during the COVID-19 pandemic, telemedicine has been paid unprecedented attention, which can break through the barriers of medical attendance caused by COVID-19 (Zampino et al., 2021). “Internet + Anticoagulation clinic” (IAC) is a new approach for anticoagulation management, which is undoubtedly attractive to patients and clinical pharmacists, especially during the COVID-19 pandemic. To the best of our knowledge, only one previous study has investigated the effectiveness and safety of anticoagulation management provided by clinical pharmacists through the IAC during the COVID-19 pandemic (Jiang et al., 2022), with small sample size and short-term follow-ups, providing preliminary conclusions that IAC improved anticoagulation quality. Therefore, more studies are encouraged to provide crucial evidence for application of IAC during the COVID-19 pandemic.

More importantly, it is necessary for clinical pharmacists to accurately identify patients with poor anticoagulation quality and intervene early, which can improve their anticoagulation quality, and no studies have focused on the prediction of anticoagulation quality during the COVID-19 pandemic until now. The time in therapeutic range (TTR) calculates the time period in which the INR is controlled within the therapeutic range, which is commonly applied as a measure of anticoagulation quality (Rosendaal et al., 1993). However, the TTR cannot measure the degree of stability of INR control, and INR variability can (Numao et al., 2017). The previous study found that using both INR variability and TTR could distinguish patients with increased risk of adverse events more accurately than using a single item (Labaf et al., 2015). Therefore, we used both INR variability and TTR to measure the anticoagulation quality and for the first time proposed using machine learning (ML) technology to develop a model for anticoagulation quality prediction. ML can incorporate enormous numbers of variables, has been successfully applied in the medical field, and has shown excellent performance.

This study aimed to investigate the role of IAC in warfarin treatment and develop an ML model for anticoagulation quality prediction to optimize anticoagulation treatment during the COVID-19 pandemic.

## Methods

### Study design and participants

This was a retrospective, observational study. Patients who received warfarin at the Nanjing Drum Tower Hospital from January 2020 to September 2021 (a period of the COVID-19 pandemic in China) were enrolled and analyzed. The study protocol was approved by the Ethics Committee of the Nanjing Drum Tower Hospital (No. 2020-029). The inclusion criteria were as follows: 1) patients who had been taking warfarin for 3 weeks for thromboprophylaxis of conditions such as atrial fibrillation (AF), deep venous thrombosis (DVT), pulmonary embolism (PE), and valvular heart disease (VHD); 2) age ≥18 years; and 3) had at least four eligible INR values, and the total follow-up time was more than 30 days. The exclusion criteria were as follows: 1) patients whose interval between any two adjacent INR was >120 days and who were considered lost to follow-up; 2) patients who had to discontinue warfarin therapy due to surgery or other reasons during the study period; and 3) patients with pregnancy, malignant tumor, or hemodialysis treatment.

Patients could choose anticoagulation management modes according to their conditions and were divided into two groups: the HAC group comprising patients who obtain AMSs through the hospital anticoagulation clinic, and the IAC group comprising patients who obtain AMSs through the “Internet + Anticoagulation clinic”. For patients in the HAC group, the INR results were derived from blood analysis at the Drum Tower Hospital. For patients in the IAC group, the INR results were derived from blood analysis at local hospitals or point-of-care test (POCT).

### Anticoagulation management modes

Specialist anticoagulation pharmacists provided AMSs through HAC and IAC for patients with chronic warfarin treatment. In order to eliminate biases from management content, both modes followed a standard interview with the same structure. Pharmacists collected relative information, including demographic characteristics, comorbidities, patient compliance, INR values, previous warfarin doses, concomitant medication, and adverse events. Then, pharmacists informed patients of warfarin dosing decisions and follow-up plans and conducted detailed medication education.

The IAC was a virtual clinic powered by the Internet and a mobile phone application (APP). Patients needed to fill in basic information when logging in to the APP and then upload photos of the INR test and answer questions about compliance, adverse events, and changes in concomitant medication since the previous test. Pharmacists would communicate with the patients through the APP conversation window and inform patients with medication education the dose of warfarin and follow-up plans.

### Data collection

Data were collected from the hospital information system and standardized anticoagulation record database administered by pharmacists. Basic information was recorded for each patient, including demographic characteristics, indications for warfarin, and location. INR values, warfarin dose, adverse events, concomitant medication, and test date were recorded at each encounter in a standardized anticoagulation record database.

### Study outcomes

The primary outcome was anticoagulation quality. Patients were considered to have good anticoagulation quality (the INR value was within the therapeutic range stably) only when they met both the criteria: TTR ≥60% and INR variability <0.65. Secondary outcomes were the incidence of adverse events, including thromboembolic and bleeding events.

The TTR was calculated using a linear interpolation method recommended by Rosendaal et al. (1993). Referring to the prevailing antithrombotic guidelines in China, the recommended therapeutic range of the INR was 2.0–3.0 for patients with AF, PE, and DVT and 1.5–2.5 for patients with VHD (diseases, 2018). INR variability was calculated using the method described by Fihn et al. (Fihn et al., 2003; van Leeuwen et al., 2008). ${INR}_{i}$ is the INR value of each test, and $\tau_{i}$ is the time interval between two INR tests (van Leeuwen et al., 2008). INR variability measured the stability of INR control by calculating the time-weighted INR variance. The study conducted by Labaf et al. (2015) confirmed that when the INR variability was ≥0.65, the risk of thromboembolism and major bleeding was significantly increased. Therefore, the patients were divided into two groups: the good anticoagulation quality group comprised patients who met both TTR ≥60% and INR variability <0.65, and the other patients were found in the poor anticoagulation quality group.

$$\sigma^{2}=\frac{1}{n-1}\overset{n}{\underset{i=2}{\sum }}\frac{{\left({INR}_{i}-{INR}_{i-1}\right)}^{2}}{\tau_{i}},\tau =\tau_{i}-\tau_{i-1}$$ (1)

Secondary outcomes were thromboembolism and bleeding events. Thromboembolism included DVT, PE, systemic embolism (SE), stroke, and transient ischemic attack (TIA) (Wallentin et al., 2010). Referring to the recommendations of the International Society of Thrombosis and Hemostasis (ISTH), bleeding events included major bleeding and clinically relevant non-major bleeding (CRNMB) (Schulman et al., 2010).

### Machine learning model development

The whole dataset was randomly assigned to a training set for training the model and a test set for evaluating the model (7:3), meaning that the ratio of patients with poor anticoagulation quality was maintained across both sets. The correlations of categorical and continuous variables with the anticoagulation quality were assessed using the chi-square test and the Wilcoxon–Mann–Whitney test, respectively (Morang’a et al., 2020). The variables with *p* < 0.05 were considered statistically significant and were included in machine learning to avoid the inference of irrelevant features (Liu et al., 2021).

To select the ML algorithm that exhibits the best predictive ability, five well-accepted ML classifiers, K-nearest neighbors (KNN), support vector machine (SVM), random forest classifier (RFC), eXtreme Gradient Boosting (XGBoost), and Light Gradient Boosting Machine (LightGBM), were implemented for model construction to predict anticoagulation quality. The synthetic minority oversampling technique (SMOTE) (Blagus and Lusa, 2013) was used to balance the unbalanced data in the training set, and then the training set was learned by five ML algorithms to construct the model with 5-fold cross-validation, and the optimal value of the hyperparameters was determined using a grid search algorithm.

The receiver operating characteristic curve (ROC) and area under the curve (AUC), sensitivity, specificity, and accuracy in the test set were calculated to evaluate the predictive performance of five ML models. Then, the Shapley Additive exPlanations (SHAP) method, a visualized approach based on game theory, was used to interpret the individual variable impacts on the ML models (Lundberg and Lee, 2017).

### Statistical analyses

Continuous variables were reported as median value and interquartile range (IQR) and were compared using the Wilcoxon–Mann–Whitney test. Categorical data were expressed as frequencies and percentages and were compared by the chi-square test or Fisher test. *p* < 0.05 was considered statistically significant. The ML algorithms were performed using *Python* 3.8 (https://www.python.org/) and the scikit-learn framework (https://www.scikit-learn.org/stable/). All statistical analyses were conducted using SPSS (version 22.0).

## Results

### Patient characteristics

**FIGURE 1:**
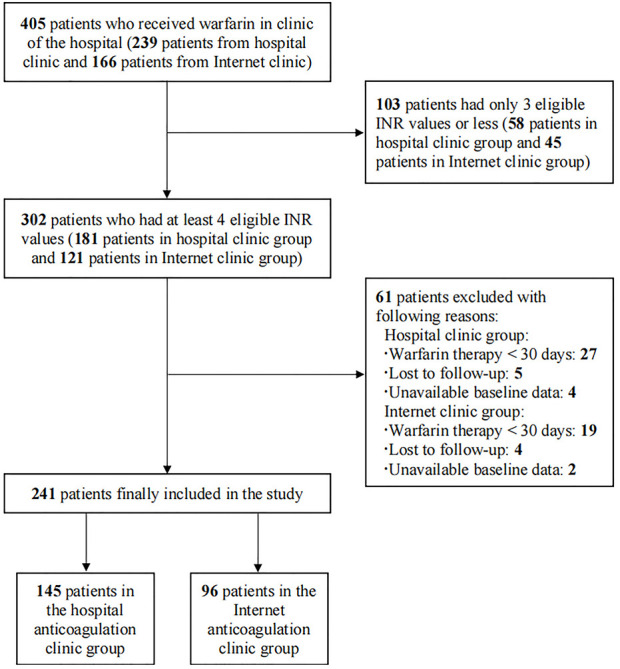
Flow diagram of the selection of patients.

A total of 405 patients who received warfarin were reviewed initially between January 2020 and September 2021, and the process of patient selection is presented in Figure 1. Finally, 241 patients were included in this study, 145 patients in the HAC group and 96 in the IAC group. Table 1 presented the demographics and characteristics of the patients. The median age of the patients was 57 years, 128 (53.1%) patients were male, and the main indication for warfarin treatment was VHD (93.8%). Hypertension (37.8%) and pulmonary arterial hypertension (39.0%) were the most common comorbidities. Beta-blockers (60.2%) were the most common concomitant medication. No significant difference was observed in baseline characteristics between the HAC and IAC groups.

**TABLE 1:** Demographics and characteristics of patients classified by anticoagulation management mode.

Characteristics	All patients (*n* = 241)	Hospital anticoagulation clinic (*n* = 145)	Internet anticoagulation clinic (*n* = 96)	*p* value
Age, years	57 (47, 66)	57 (46, 66)	56 (48, 66)	0.848
Male, n (%)	128 (53.1)	78 (53.8)	50 (52.1)	0.795
BMI (kg/m^2^)	22.9 (20.5, 25.2)	23.2 (20.8, 25.8)	22.0 (20.3, 24.8)	0.061
INR therapeutic range, n (%)				
1.5–2.5	226 (93.8)	135 (93.1)	91 (94.8)	0.595
2.0–3.0	15 (6.2)	10 (6.9)	5 (5.2)	
Education, n (%)				0.280
Primary school and below	78 (32.4)	42 (29.0)	36 (37.5)	
Middle school and above	163 (67.6)	103 (71.0)	60 (62.5)	
Comorbidities, n (%)				
Hypertension	91 (37.8)	54 (37.2)	37 (38.5)	0.838
Diabetes	13 (5.4)	8 (5.5)	5 (5.2)	0.917
Coronary artery disease	34 (14.1)	19 (13.1)	15 (15.6)	0.582
Pulmonary arterial hypertension	94 (39.0)	52 (35.9)	42 (43.8)	0.219
Renal insufficiency	18 (7.5)	8 (5.5)	10 (10.4)	0.157
History of thromboembolism	7 (2.9)	4 (2.8)	3 (3.1)	1.000
History of stroke	23 (9.5)	14 (9.7)	9 (9.4)	0.942
History of hemorrhage	3 (1.2)	1 (0.7)	2 (2.1)	0.717
Medications, n (%)				
Aspirin	23 (9.5)	15 (10.3)	8 (8.3)	0.603
Amiodarone	22 (9.1)	11 (7.6)	11 (11.5)	0.307
Digoxin	46 (19.1)	25 (17.2)	21 (21.9)	0.370
ACEI/ARB	28 (11.6)	15 (10.3)	13 (13.5)	0.448
Beta-blockers	145 (60.2)	91 (62.8)	54 (56.3)	0.312
Statins	44 (18.3)	32 (22.1)	12 (12.5)	0.060

BMI, body mass index; INR, international normalized ratio; ACEI, angiotensin-converting enzyme inhibitors; ARB, angiotensin receptor blocker.

### Anticoagulation quality of patients in HAC and IAC

The TTR, INR variability, and adverse events of 241 patients with 1652 INR values were calculated (Table 2). 106 (73.1%) and 67 (69.8%) patients had good anticoagulation quality in the HAC group and the IAC group, and the average TTR was 79.9 ± 20.0% and 80.6 ± 21.1%, respectively. The average TTR of the 241 patients included in this study was 80.2 ± 20.4%. During the follow-up period, five patients (2.07%) experienced thromboembolic or major bleeding events and needed hospital admission care, and the remaining suffered only minor bleeding. No significant difference in the incidences of major bleeding (0.69 vs. 1.0%, *p* = 1.000), CRNMB (39.3 vs. 40.6%, *p* = 0.838), and thromboembolic (1.4 vs. 1.0%, *p* = 1.000) was detected between patients in the HAC group and the IAC group.

**TABLE 2:** Comparison of clinical outcomes of Hospital anticoagulation clinic vs. Internet anticoagulation clinic.

Clinical outcomes	All patients (*n* = 241)	Hospital anticoagulation clinic (*n* = 145)	Internet anticoagulation clinic (*n* = 96)	*p* value
Good anticoagulation quality	173 (71.8)	106 (73.1)	67 (69.8)	0.576
TTR (%)	80.2 ± 20.4	79.9 ± 20.0	80.6 ± 21.1	0.644
TTR ≥60%, n (%)	203 (84.2)	122 (84.1)	81 (84.4)	0.961
INR variability ≥0.65	46 (19.1)	26 (17.9)	20 (20.8)	0.575
Major bleeding, n (%)	2 (0.83)	1 (0.69)	1 (1.0)	1.000
CRNMB, n (%)	96 (39.8)	57 (39.3)	39 (40.6)	0.838
Oral hemorrhage	40 (16.6)	22 (15.2)	18 (18.8)	0.465
Epistaxis	20 (8.3)	15 (10.3)	5 (5.2)	0.157
Subconjunctival bleeding	10 (4.1)	6 (4.1)	4 (4.2)	1.000
Subcutaneous bleeding	12 (5.0)	4 (2.8)	8 (8.3)	0.100
Gastrointestinal bleeding	7 (2.9)	6 (4.1)	1 (1.0)	0.313
Hematuria	5 (2.1)	3 (2.1)	2 (2.1)	1.000
Metrorrhagia	2 (0.83)	1 (0.69)	1 (1.0)	1.000
Thromboembolic events, n (%)	3 (1.2)	2 (1.4)	1 (1.0)	1.000
Peripheral artery thrombosis	1 (0.4)	0 (0.0)	1 (1.0)	0.398
Valve thrombosis	1 (0.4)	1 (0.69)	0 (0.0)	1.000
Stroke	1 (0.4)	1 (0.69)	0 (0.0)	1.000

INR, international normalized ratio; TTR, time in therapeutic range; CRNMB, clinically relevant non-major bleeding.

### Machine learning models for anticoagulation quality prediction

The 241 patients were classified according to anticoagulation quality ( good anticoagulation quality group and poor anticoagulation quality group). The baseline characteristics of the two groups are shown in Table 3. The whole dataset was randomly assigned to a training set and a test set, and the baselines of the two sets were relatively balanced (Supplementary Table S1). According to variable selection, seven variables were significantly correlated with the chronic anticoagulation quality in the training set, including age, education, hypertension, renal insufficiency, and combined use of aspirin, amiodarone, and statins (Supplementary Table S2). Heatmap visualization of the correlations between the anticoagulation quality and the variables in the training set is shown in Supplementary Figure S1.

**TABLE 3:** Demographics and characteristics of patients classified by anticoagulation quality.

Characteristics	Good anticoagulation quality (*n* = 173)	Poor anticoagulation quality (*n* = 68)	*p* value
Age, years	54 (42.5, 63)	65 (55.3, 71)	0.000*
Male, n (%)	93 (53.8)	35 (51.5)	0.749
BMI (kg/m^2^)	22.8 (20.3, 25.3)	23.1 (21.1, 25.1)	0.454
Education, n (%)			0.000*
Primary school and below	41 (23.7)	37 (54.4)	
Middle school and above	132 (76.3)	31 (45.6)	
Comorbidities, n (%)			
Hypertension	57 (32.9)	34 (50.0)	0.014*
Diabetes	6 (3.5)	7 (10.3)	0.073
Coronary artery disease	23 (13.3)	11 (16.2)	0.563
Renal insufficiency	8 (4.6)	10 (14.7)	0.007*
Pulmonary arterial hypertension	64 (37.0)	30 (44.1)	0.308
History of thromboembolism	4 (2.3)	3 (4.4)	0.655
History of stroke	16 (9.2)	7 (10.3)	0.804
History of hemorrhage	1 (0.6)	2 (2.9)	0.193
Medications, n (%)			
Aspirin	10 (5.8)	13 (19.1)	0.002*
Amiodarone	9 (5.2)	13 (19.1)	0.001*
Digoxin	29 (16.8)	17 (25.0)	0.143
ACEI/ARB	17 (9.8)	11 (16.2)	0.166
Beta-blockers	107 (61.8)	38 (55.9)	0.394
Statins	26 (15.0)	18 (26.5)	0.039*

*Univariate analysis showed significant difference between the two group.

BMI, body mass index; INR, international normalized ratio; ACEI, angiotensin-converting enzyme inhibitors; ARB, angiotensin receptor blocker.

**FIGURE 2:**
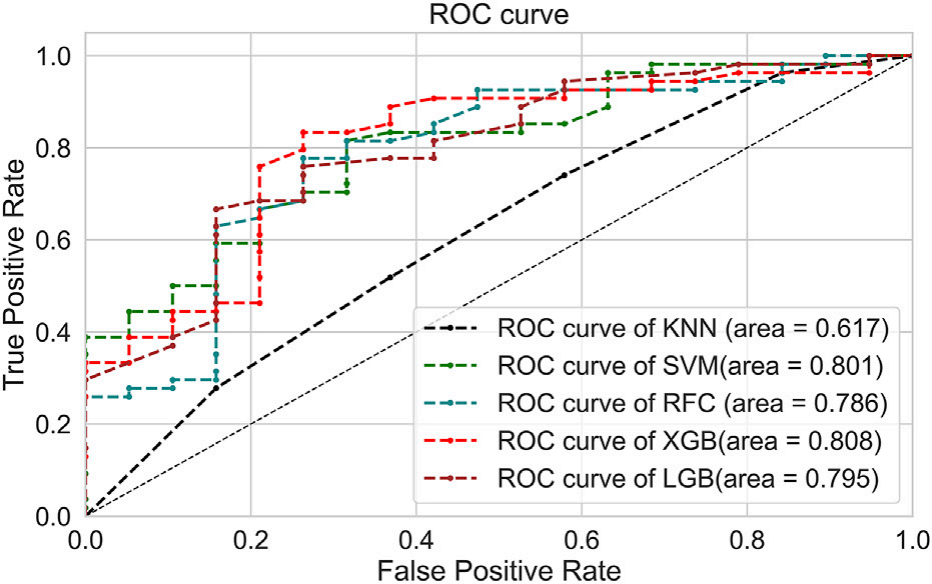
Receiver operating characteristic curve of the ML models.

Five ML models were developed using seven selected variables on the training set to predict anticoagulation quality. The optimal value for the hyperparameters with a grid search algorithm is shown in Supplementary Table S3, and the results for five folds are shown in Supplementary Table S4. Then, the test set was used to test the predictive performance of each model, and the results are shown in Table 4 and Figure 2. Although the RFC model has the best specificity among the five models, the high sensitivity is particularly critical given that model is designed to recognize more patients with poor anticoagulation quality, and the XGBoost model is the optimum model with the consideration of the best sensitivity (76.7%), AUC (0.808), and accuracy (0.767).

**TABLE 4:** Prediction performance of the five machine learning models on the test set.

Machine learning model	AUC	Sensitivity	Specificity	Accuracy
KNN	0.617	0.6316	0.5185	0.548
SVM	0.801	0.790	0.593	0.644
RFC	0.786	0.737	0.778	0.767
XGBoost	0.808	0.790	0.759	0.767
LightGBM	0.795	0.737	0.685	0.699

**FIGURE 3:**
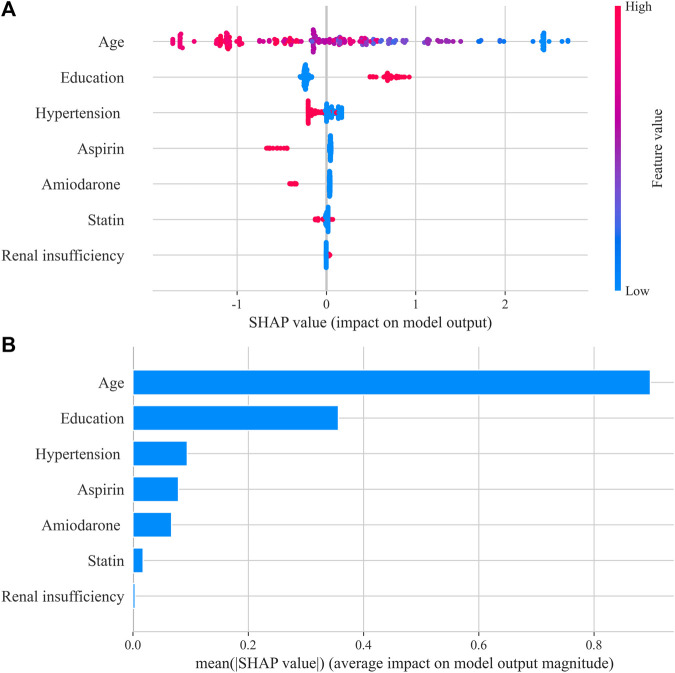
Shapley Additive exPlanations (SHAP) summary plot in the XGBoost model. **(A)** SHAP beeswarm plot showed the distribution of SHAP values of each feature. Red represents higher feature values, and blue represents lower feature values. **(B)** Typical bar chart of feature importance was shown based on the mean absolute SHAP value of each feature.

The SHAP method was used to interpret the optimum ML model (XGBoost) and provided a direct visual understanding of feature contributions. The results suggested that age, education, hypertension, aspirin, and amiodarone were the top five important features, as shown in Figure 3. Poor anticoagulation quality was a high probability with patients of older age, low education, hypertension, aspirin, and amiodarone.

## Discussion

In this study, the Internet and machine learning techniques were used for the first time to optimize warfarin anticoagulation management during the COVID-19 pandemic in China. The role of the IAC in the care of patients who received warfarin during the COVID-19 pandemic was proven, and no significant difference in anticoagulation quality and incidence of adverse events was detected between patients in the IAC and HAC groups. The IAC could break the difficulties of medical treatment due to COVID-19-induced lockdowns, which was an important healthcare approach during the COVID-19 pandemic. Furthermore, we used both INR variability and TTR to measure the anticoagulation quality and for the first time developed an ML model (XGBoost) to predict anticoagulation quality and make individual decisions regarding who could benefit from active intervention.

The universally adopted strategy of reducing social interaction during the COVID-19 pandemic has led to unprecedented difficulties in the ongoing healthcare of chronically ill patients (Kow et al., 2020; Prem et al., 2020). A new approach to patient care was demanded. In this study, patients in the IAC group had a similar anticoagulation quality to those in the HAC group, and both groups had good anticoagulation quality. The average TTR of patients in the two groups was 79.9 ± 20.0% and 80.6 ± 21.1%, respectively. A meta-analysis pooling 95 studies worldwide reported that the patients were only 61% of the time in the therapeutic range (Mearns et al., 2014), and the TTR of patients managed in our institution was better than this level. It was interesting to note that patients in the HAC group achieved good anticoagulation quality even during the COVID-19 pandemic, which was consistent with the study conducted by Cope et al. (2021). However, patients in the HAC group needed to pay more time and money to obtain AMSs than before the COVID-19 pandemic and were at risk of exposure to COVID-19. The use of telemedicine for patient care, such as IAC, ensured social distancing and reduced person-to-person contact. It provided convenient access to routine care with low risk of virus transmission (Al Ammari et al., 2021).

High-quality anticoagulation was the key to ensure efficacy and safety of warfarin administration (Singer et al., 2013). Therefore, it is important for clinical pharmacists to identify patients with poor anticoagulation quality accurately and carry out interventions for these patients early, especially during the COVID-19 pandemic. We applied an ML algorithm to develop a prediction model of warfarin anticoagulation quality in the Chinese population for the first time. The XGBoost is an ensemble algorithm based on a tree-like structure, which combines multiple individual weak prediction models to produce a robust predictive estimator (Huang et al., 2021) and showed good predictive performance in this study, with AUC = 0.808 and accuracy = 0.767. Several studies have been conducted, such as the SAME-TT₂R₂ score (Apostolakis et al., 2013) and the Nomogram model established by Wang et al. (2021), both of which were used to predict the anticoagulation quality in AF patients, with a moderate predictive ability (c-index of SAME-TT₂R₂ = 0.72; c-index of the Nomogram model = 0.718). However, a single TTR value was used in both studies to evaluate the anticoagulation quality, and TTR could not measure the degree of stability of INR control, resulting in the limitation of anticoagulation quality evaluation (Wang et al., 2021). Even if the patient had a high TTR, the unstable anticoagulation intensity would lead to poor prognosis, and INR variability could compensate for this deficiency. Therefore, patients with a high TTR and low INR variability were defined as those with good anticoagulation quality in this study, whose INR value remained stable within the therapeutic range. The better anticoagulation quality standard and more accurate prediction ability than that of the SAMe-TT₂R₂ score and the Nomogram model gave the XGBoost model considerable value for clinical application.

It is crucial for medical staff to understand how the model predicts risk for patients (Zheng et al., 2022). In this study, the risk factors associated with poor anticoagulation quality were ranked and provided a visual interpretation by the SHAP method. Age was the driving predictor of poor anticoagulation quality, which was also an important predictor of the SAME-TT₂R₂ model (Apostolakis et al., 2013) and the nomogram model (Wang et al., 2021). In elderly patients, the decline in self-management ability, multiple additional complications, and multiple medications was common, which would inevitably impact the efficacy of warfarin (Qiu et al., 2020). In addition, patients with low educational background were associated with poor anticoagulation quality and had insufficient understanding of the importance of warfarin therapy and insufficient knowledge of anticoagulation, resulting in a lack of self-management ability (Rodriguez et al., 2013). Therefore, medication education was crucial for these patients, which should be focused on by clinical pharmacists in clinical work. Hypertension and renal insufficiency were also associated with the poor quality of anticoagulation, which has been interpreted in previous studies. Renal insufficiency was associated with poor anticoagulation quality in Americans (Pokorney et al., 2015), and hypertension contributed to poor anticoagulation quality in African patients (Mwita et al., 2018). In addition, concomitant medications, such as amiodarone and aspirin, were found to be indicators of poor INR control. Amiodarone competes with warfarin for cytochrome P450 2C9, which is the major enzyme of warfarin metabolism, leading to fluctuations in anticoagulation intensity and even major bleeding events (Holm et al., 2017). Interactions of aspirin and warfarin on different components of the coagulation pathway can increase the risk of bleeding (Proietti and Lip, 2018). Although some factors are the inherent diseases and necessary drug treatment of patients which are hard to change, measures such as strengthening medication education can effectively improve the anticoagulation quality of patients. Previous studies showed that standardized medication education by pharmacists could significantly improve the quality of anticoagulation and also reduced the number of emergency admission (Verret et al., 2012; Neshewat et al., 2021).

Overall, the XGBoost model is expected to be applied in practice to select patients who could benefit from active intervention to improve warfarin treatment during the COVID-19 pandemic.

## Limitations

There are several limitations to this study. First, this was a retrospective, observational study that might introduce selection bias. Second, patients in the IAC group were allowed to get the INR results from blood analysis at local hospitals, although patients could simplify the process of medical care in the local hospital, person-to-person contact could not be completely avoided, and POCT is a novel method for anticoagulation management during the COVID-19 pandemic. Third, some variables have not been collected, such as the patients’ smoking or alcohol intake and patient genotypes (VKORC1 and CYP2C9), which limited the analysis, although patients with smoking and alcohol abuse are rare in our clinical practice. Finally, although the current study supports to apply machine learning models to predict anticoagulation quality as a decision-support technology, external validations are necessary.

## Conclusion

In the real-world setting of our hospital, “Internet + Anticoagulation clinic” played a positive role in warfarin treatment during the COVID-19 pandemic, which ensured good anticoagulation quality and decreased person-to-person contact, with a low risk of virus transmission. Furthermore, the ML model offers a new avenue to select patients at high risk of poor anticoagulation quality, which can improve the warfarin therapy decision-making during the COVID-19 pandemic.

## Data Availability

The data that support the finding of this study is available on request from the corresponding author. The data are not publicly available due to ethical restrictions.
