# Supplementary material for: Warfarin anticoagulation management during the COVID-19 pandemic: The role of internet clinic and machine learning
Source: Front Pharmacol. 2022 Sep 26;13:933156. doi: 10.3389/fphar.2022.933156 (PMC9549053; doi:10.3389/fphar.2022.933156)
Supplement: Supplementary file 1 [file Table1.DOCX]

Supplementary Materials

**Supplementary Table S1.** Demographics and characteristics of patients classified by training set and test set.

| Characteristics | Training set (n=168) | Test set (n=73) | *P* Value |
| --- | --- | --- | --- |
| Age, years | 56 (47, 67) | 58 (48, 65) | 0.905 |
| Male, n (%) | 86 (51.2) | 42 (57.7) | 0.364 |
| BMI (kg/m^2^) | 23.1 (20.5, 25.3) | 22.3 (20.6, 25.1) | 0.459 |
| Education, n (%) |  |  | 0.277 |
| Primary school and below | 58 (34.5) | 20 (27.4) |  |
| Middle school and above | 110(65.5) | 53(72.6) |  |
| Comorbidities, n (%) |  |  |  |
| Hypertension | 67 (39.9) | 24 (32.9) | 0.303 |
| Diabetes | 9 (5.4) | 4 (5.5) | 1.000 |
| Coronary artery disease | 23 (13.7) | 11 (15.1) | 0.778 |
| Renal insufficiency | 13 (7.7) | 5 (6.8) | 0.809 |
| Pulmonary arterial hypertension | 71 (42.3) | 23 (31.5) | 0.116 |
| History of thromboembolism | 4 (2.4) | 3 (4.1) | 0.751 |
| History of stroke | 14 (8.3) | 9 (12.3) | 0.332 |
| History of hemorrhage | 2 (1.2) | 1 (1.4) | 0.908 |
| Medications, n (%) |  |  |  |
| Aspirin | 15 (8.9) | 8 (11.0) | 0.622 |
| Amiodarone | 18 (10.7) | 4 (5.5) | 0.195 |
| Digoxin | 32 (19.0) | 14 (19.2) | 0.981 |
| ACEI/ARB | 19 (11.3) | 9 (12.3) | 0.821 |
| Beta-blockers | 102 (60.7) | 43 (58.9) | 0.792 |
| Statins | 30 (17.9) | 14 (19.2) | 0.807 |
| Good anticoagulation quality | 119 (70.8) | 54 (74.0) | 0.619 |

BMI, Body Mass Index; INR, International Normalized Ratio; ACEI, angiotensin-converting enzyme inhibitors; ARB, angiotensin receptor blocker.

**Supplement Table S2.** The correlations of the variables with the anticoagulation quality in the training set

| Continuous variables | *P* | Categorical variables | *P* |
| --- | --- | --- | --- |
| Age | 0.000 | Education | 0.005 |
|  |  | Hypertension | 0.025 |
|  |  | Renal insufficiency | 0.042 |
|  |  | Aspirin | 0.006 |
|  |  | Amiodarone | 0.002 |
|  |  | Statins | 0.020 |


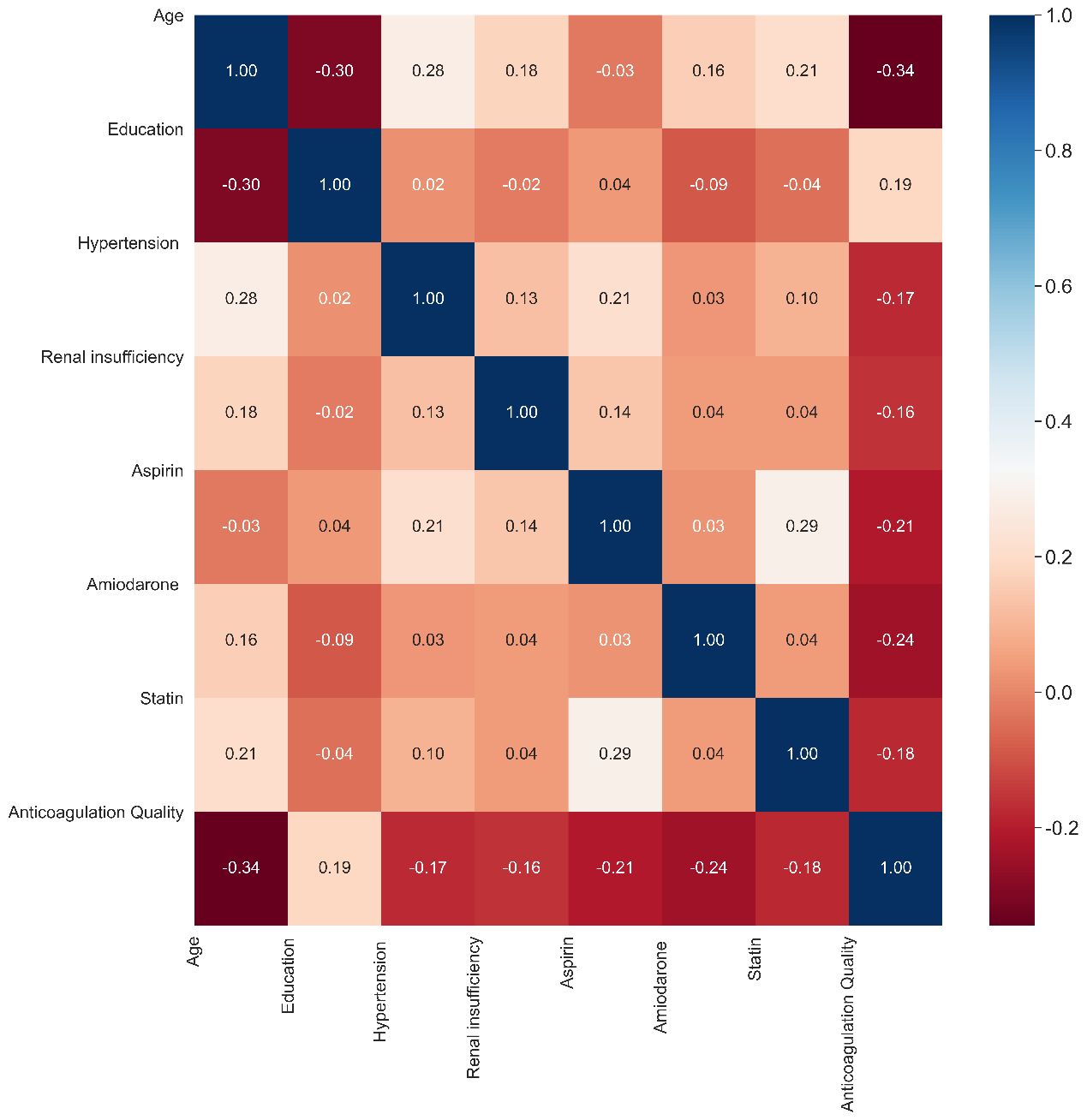


**Supplement Figure S1.** Heatmap visualization of the correlations between the anticoagulation quality and the variables in the training set.

**Supplement Table S3.** The hyperparameters of five ML models.

| Model | Hyper-parameters | Value |
| --- | --- | --- |
| KNN | N Neighbors | 4 |
| SVM | C | 1 |
|  | Gamma | 0.01 |
| RFC | N estimators | 130 |
|  | Max depth | 5 |
|  | Min samples leaf | 3 |
|  | Min samples split | 12 |
| XGBoost | Max depth | 3 |
|  | Learning rate | 0.07 |
|  | N estimators | 80 |
|  | Min child weight | 2 |
|  | Gamma | 0.15 |
|  | Subsample | 0.70 |
|  | Colsample bytree | 0.6 |
|  | Reg alpha | 0.01 |
|  | Reg lambda | 0.01 |
| LightGBM | Max depth | 3 |
|  | Learning rate | 0.05 |
|  | N estimators | 80 |
|  | Bagging fraction | 0.8 |
|  | Bagging freq | 2 |
|  | Feature fraction | 0.6 |
|  | Num leaves | 3 |

**Supplement Table S4.** The results for all folds in 5-fold cross-validation.

| Folds | AUC | Accuracy |
| --- | --- | --- |
| 1 | 0.822 | 0.75 |
| 2 | 0.819 | 0.71 |
| 3 | 0.725 | 0.66 |
| 4 | 0.786 | 0.68 |
| 5 | 0.687 | 0.58 |
